# MEASURING UNMET NEEDS FOR COMMUNITY-DWELLING CONSUMERS OF HOME- AND COMMUNITY-BASED SERVICES USING NCI-AD DATA

**DOI:** 10.1093/geroni/igad104.0806

**Published:** 2023-12-21

**Authors:** Tetyana Shippee, Taylor Bucy, John Mulcahy, Eric Jutkowitz

**Affiliations:** University of Minnesota, Minneapolis, Minnesota, United States; University of Minnesota School of Public Health, Minneapolis, Minnesota, United States; University of Minnesota, Minneapolis, Minnesota, United States; Brown University, Providence, Rhode Island, United States

## Abstract

Rebalancing efforts have focused on helping more people age in the community rather than institutional settings. Yet, we know little of whether actual services being offered meet consumer needs. HCBS provides intermittent services and supports to consumers and variability can create increased reliance on informal caregivers, contributing to unmet need. This can be especially true for consumers living with Alzheimer’s disease and Alzheimer’s disease and related dementias (AD/ADRD), whose needs may fluctuate and intensify as functional and/or cognitive impairments advance. This presentation examines unmet need among a national sample of older adult (65+) consumers of publicly funded HCBS, using data from the National Core Indicators - Aging and Disability (NCI-AD) survey from 2016-2019. We restrict our sample to community-dwelling older adults, where community-dwelling includes: own or family house/apartment, senior living apartment/complex, group home, adult family home, foster, or host home. Our results showed that services that were most commonly used were home-based services (57%), with at least half of the consumers using two different services to support their needs. Factors associated with wanting more services included being a woman, living alone, having a physical disability, and a diagnosis of AD/ADRD. Older adults with AD/ADRD desired more services than those without AD/ADRD, with additional desired services for caregiver supports and transportation. Our findings speak to the need to measure and improve person-centered quality of publicly-funded HCBS for older adults, with particular attention to the needs of those living with AD/ADRD.

